# A Risk Function for Behavioral Disruption of Blainville’s Beaked Whales (*Mesoplodon densirostris*) from Mid-Frequency Active Sonar

**DOI:** 10.1371/journal.pone.0085064

**Published:** 2014-01-21

**Authors:** David Moretti, Len Thomas, Tiago Marques, John Harwood, Ashley Dilley, Bert Neales, Jessica Shaffer, Elena McCarthy, Leslie New, Susan Jarvis, Ronald Morrissey

**Affiliations:** 1 Naval Undersea Warfare Center, Newport, Rhode Island, United States of America; 2 Centre for Research into Ecological and Environmental Modelling, University of St Andrews, St. Andrews, Scotland; 3 U.S. Marine Mammal Commission, Bethesda, Maryland, United States of America; Texas A&M University-Corpus Christi, United States of America

## Abstract

There is increasing concern about the potential effects of noise pollution on marine life in the world’s oceans. For marine mammals, anthropogenic sounds may cause behavioral disruption, and this can be quantified using a risk function that relates sound exposure to a measured behavioral response. Beaked whales are a taxon of deep diving whales that may be particularly susceptible to naval sonar as the species has been associated with sonar-related mass stranding events. Here we derive the first empirical risk function for Blainville’s beaked whales (*Mesoplodon densirostris*) by combining *in situ* data from passive acoustic monitoring of animal vocalizations and navy sonar operations with precise ship tracks and sound field modeling. The hydrophone array at the Atlantic Undersea Test and Evaluation Center, Bahamas, was used to locate vocalizing groups of Blainville’s beaked whales and identify sonar transmissions before, during, and after Mid-Frequency Active (MFA) sonar operations. Sonar transmission times and source levels were combined with ship tracks using a sound propagation model to estimate the received level (RL) at each hydrophone. A generalized additive model was fitted to data to model the presence or absence of the start of foraging dives in 30-minute periods as a function of the corresponding sonar RL at the hydrophone closest to the center of each group. This model was then used to construct a risk function that can be used to estimate the probability of a behavioral change (cessation of foraging) the individual members of a Blainville’s beaked whale population might experience as a function of sonar RL. The function predicts a 0.5 probability of disturbance at a RL of 150dBrms re µPa (CI: 144 to 155) This is 15dB lower than the level used historically by the US Navy in their risk assessments but 10 dB higher than the current 140 dB step-function.

## Introduction

With the continued rise in world population and the associated increase in global industrialization, the input of anthropogenic noise into the world’s oceans is of growing concern [1], [2]. Marine mammals are heavily reliant on sound for feeding, movement, and social interactions. Exposure to anthropogenic noise may therefore disrupt their behavior, with potential consequences for their health, survival, and ability to reproduce [2], [3].

If such consequences are to be managed effectively, we need to relate the effects of this potential disturbance to the overall health of the population. One of the first steps in this process is to establish the relationship between the probability of a behavioral response and the level of acoustic disturbance to which an individual is exposed. Typically, such a dose response relationship or risk function is used to assign a probability of adverse effect to a given level of exposure [4]. Determining the functional form of a contaminant’s effect on terrestrial species is difficult [5], while for marine species it is an even more daunting task.

Despite growing concern, an ever-increasing number of sound sources with the potential to affect marine mammal species are being deployed in the marine environment. Examples of these are seismic air guns, shipping, echo-sounders, pile driving, navy sonars, tools for fisheries which include various pingers, and coastal activities (ambient noise from harbors, industries, towns). Sonar has been associated with a number of cetacean mass strandings and is therefore of particular concern [6], [7], [8]. Cuvier’s (*Ziphius cavirostis*) and Blainville’s (*Mesoplodon densirostris*) beaked whales, hereafter *Zc* and *Md,* respectively, are the species that have been most frequently associated with sonar related strandings [6], [7]. Such occurrences suggest that, at certain exposure levels, these species react to sonar in a manner that goes beyond harassment and may result in physical harm [9].

The apparent sensitivity of marine mammals to anthropogenic noise has garnered increased attention from regulators, particularly in the U.S. where legal authorization to conduct operations with loud sources of underwater sound requires a prediction of the number of animals that may be affected. This mandatory prediction is usually made using so-called “effect models”. These models estimate sound exposure on individuals within a population of animals in a bounded area and predict the number of animals that are “harassed”, based on published exposure criteria [3], [10], [11], [12]. An animal’s response to sound depends on a complex mix of factors in addition to the received level of sound such as the shape of the signal (transitory short to continuous), signal bandwidth, and the animals hearing bandwidth. However, while the models may incorporate these factors, they typically depend heavily on a risk function that maps the probability of disturbance to a received level of sound and is used to assess the effect of each sound exposure. Until now, such risk functions were derived using data from captive animals and proxy species [3]. Thus, the functions that have been used to estimate the risk for sonar-sensitive beaked whales are not wholly representative of the species’ response to sound.

Historically, the U.S Navy assessed the onset of behavioral disturbance in beaked whales using a risk function derived from data for killer whales (*Orcinus orca*) exposed to Mid-Frequency Active (MFA) sonar in the Haro Strait [13], studies of captive bottlenose dolphins (*Tursiops truncatus*) [14], and controlled exposure experiments with North Atlantic right whales (*Eubalaena glacialis* ) [15]. In the light of a series of recent studies of beaked whales [16], [17], in 2012 this risk function was replaced with a step function at a received level (RL) of 140 dB re 1 µPa (root mean squared [rms]), hereafter RL_rms_ and dB respectively. Tyack *et al.* [17] measured the reaction of two beaked whales tagged at the Atlantic Undersea Test and Evaluation Center (AUTEC) in the Bahamas and exposed to a signal from a 210 dB re 1 µPa @ 1m source that resembled MFA sonar and was positioned within 3 km of the animals. The exposed animals terminated their foraging dives and then moved slowly towards the surface and away from the source [17]. This occurred at a RL_rms_ of approximately 138 dB. Moretti *et al*. [18] showed that *Md* abundance within AUTEC declined from 22 animals (95% confidence interval (CI): 17–28) before a multi-ship MFA operation, to six animals (4–8) during, and then increased to 32 (CI: 25–40) after the cessation of sonar. McCarthy *et al.* [16] exploited the vocal behavior of *Md* [19], which execute deep foraging dives as a group and click only at depth (>300 m) during these dives. The detection of *Md* clicks was used as a proxy for diving groups of animals. By detecting vocal groups of *Md,* they were able to document population level movement in response to a MFA sonar operation. 4.04 *Md* vocal groups per hour (CI: 3.81–4.27) were detected in a 65 hour period prior to a sonar operation. This estimate dipped to 1.36 vocal groups per hour (CI: 1.05–1.67) during 68 hours of sonar operations. During this same time period, only 17 groups were detected coincident with sonar tranmissons at a mean RL_rms_ of 128 dB (120.9–135.1). The majority of groups vocalized while ships were repositioning and were not tranmitting sonar.

In this paper, we develop a new behavioral risk function for Blainville’s beaked whale exposure to MFA sonar based on empirical data collected at AUTEC where the species is regularly detected [18]. AUTEC is located in the Tongue of the Ocean (TOTO) which forms the southern branch of the Great Bahama Canyon and is connected to the Northwest Providence Channel, where one of the most studied mass stranding events occurred on 21 April, 2000. In that event nine *Zc*, four *Md,* two unidentified beaked whales, and two minke whales (*Balaenoptera acutorostrata*) stranded on the surrounding islands during a MFA sonar operation in which five ships systematically moved from East to West in an Anti-Submarine Warfare (ASW) choke point exercise [8], [20]. Despite striking similarities in bathymetry and routine use of MFA sonar, no mass strandings have been reported at AUTEC.

Past studies, conducted at AUTEC [16], [17], [18], document *Md* reactions to sonar, but did not provide sufficient data to define the risk of behavioral disturbance as a function of exposure level. To derive the risk function, archival records from AUTEC hydrophones collected during multi-ship sonar operations were examined to identify vocalizing *Md* groups and sonar pings. The sonar pings were then associated with the precise locations of the transmitting ships, and the combined data were used in a propagation model to estimate the whales’ sound exposure levels. The probability of initiating a foraging dive with no sonar present was compared with that measured in the presence of sonar during an MFA operation to produce the first risk function for *Md.*

## Methods

### Data

The Marine Mammal Monitoring on Navy Ranges (M3R) program has developed a set of passive acoustic tools for *in situ* monitoring of cetaceans on U.S. Navy undersea ranges. The AUTEC range is designed for the testing and evaluation of Navy systems and for anti-submarine warfare training. It is composed of a large array of 91 bottom-mounted hydrophones. The range layout is optimized to track undersea vehicles that emit a known signal at a frequency of approximately 37 kHz and source level of approximately 194 dB re 1 µPa @ 1 m, at a known repetition rate. Given their designed frequency response and sensitivity, the hydrophones can also be used to detect, classify, and localize marine mammals, like beaked whales, which are known to vocalize around this frequency and have a measured source level in excess of 200 dB re 1 µPa @ 1 m [21]. Echolocation clicks produced by groups of beaked whales are routinely detected on the AUTEC range [18], [22].

Two separate data sets were used in the analysis. The first consisted of acoustic detection archives derived from range hydrophone data. The archives contained detection reports with the output of a frequency domain energy detector, along with the precise time (<15 msec) of each click detection. The energy detector is based on a 2048 point Fast Fourier Transform (FFT) with rectangular window and 50% overlap. An adaptive threshold is applied to each bin of the FFT to generate a “detection spectrum” where all FFT bins above threshold are assigned a magnitude of 1 and 0 otherwise. These detection spectra were used to identify both groups of vocalizing animals and sonar pings as received on individual range hydrophones during actual multi-ship MFA sonar operations. The second dataset consisted of precise Global Position System (GPS) based ship tracks obtained from AUTEC, which were recorded during coincident MFA operations.

Group dive starts and the hydrophone central to the group were identified. To gather these data, *Md* vocalizations were first detected on the set of range hydrophones surrounding the group of animals. An automated procedure was used to associate clicks into click trains, and click trains associated across hydrophones to identify Group Vocal Periods (GVP). A GVP is associated with the vocal period of the dive of a group. Given AUTEC and *Md* characteristics, it can be safely assumed all dives are detected in this way. GVP start and stop times were recorded [23]. The mean Inter-Click Interval (ICI) was calculated and used as a feature for classification [16]. The center position of each group was further refined as the mean of the hydrophone locations, weighted by the number of clicks present on each group-associated hydrophone. The hydrophone closest to the mean was designated the center hydrophone.

The second data set consisted of MFA sonar pings which were visually identified in M3R detection archives and used to determine the times when ships were actively transmitting. These transmission times were compared with GPS ship tracks obtained from the AUTEC range. Based on the ship’s position and the pattern and intensity of the sonar detected on the surrounding range hydrophones (evaluated by visual inspection of the archived spectrograms), the start and stop times of all MFA sonar transmissions were established for each ship participating in the operations. The type of sonar used, its frequency, and repetition rate were determined.

The data analyzed here were obtained from a multi-ship (3 active surface ships) MFA sonar operation in May 2009. They were archived during the 19 hour period immediately before the operation and during the three days of active transmissions. The operation consisted of six distinct periods of active sonar, referred to as scenarios, which ranged in duration from 6.73 to 9.83 hours (Table 1). During each scenario the ships would seek out a silent underwater target using active sonar. It is assumed that with a single target and animals spread over 500 nm^2^, the silent target is not a factor in the animals’ responses. At the end of each scenario, the ships would reposition, generally on the southern or northern edge of the range. While repositioning, no sonar was transmitted, thus these are referred to as “gaps” in active transmission. This resulted in six scenarios with intense sonar usage separated by silent gaps of approximately 3 to 7 hours.

**Table 1:** The start and stop times of six sonar scenarios during a multi-ship exercise on the AUTEC range in 2009 with the duration of gap periods with no sonar.

Period	Start Data	Time MFA Active(hrs:min)	Duration (hrs)
Pre-Test	13-May		19.58
Scenario 1	14-May	10:47–19:56	9.15
Gap 1			4.35
Scenario 2	15-May	00:17–09:35	9.30
Gap 2			2.62
Scenario 3	15-May	12:12–21:02	9.83
Gap 3			3.65
Scenario 4	16-May	00:41–07:25	6.73
Gap 4			6.62
Scenario 5	16-May	14:02–21:57	7.92
Gap 5			4.05
Scenario 6	17-May	02:00–10:44	8.73
Post test			12.57

Operational Navy security precluded direct recording of the hydrophones during operations. In addition, the hydrophones are at a mean depth of approximately 1,700 m. The model considers the presence or absence of an *Md* dive start. Thus an animal’s decision to dive occurs at depth above 200 m and consequently the receive level of interest is within this depth regime, vice at the depth of the hydrophone. Therefore, the received level of the sonar on the center hydrophone associated with each identified *Md* group was estimated using the U.S. Navy’s acoustic effects model [24]. The model employs the Comprehensive Acoustic Simulation System Gaussian Ray Bundle (CASS/GRAB) model [25] to calculate sound propagation loss using known source levels and beam patterns for sources active during the operation. Environmental inputs to the model were obtained from the Oceanographic and Atmospheric Master Library [26], which includes bathymetry, sound speed profiles, bottom loss information, and wind speed. A 3-D seasonal sound speed profile with quarter degree resolution and a seasonal wind speed with one degree resolution were used. Modeling was done with a range step of 50 m and a depth resolution of 25 m.

A total of 18 acoustic analysis points were distributed over the range in six rows of three. In each row, one point was placed in the middle and the other two at the eastern and western boundaries of the range. Range dependent propagation loss along 18 equally spaced (20 degrees) radial axes at each analysis point was pre-calculated. For each sonar transmission, the analysis point closest to the ship’s position was translated to the location of the sonar ping transmission. The received level of a sonar ping at each hydrophone was calculated using the predicted propagation loss along the closest radial axis. *Md* spend most of their time within 200 m of the surface [27], [28], so the modeled RLs at 100 m were used on the assumption that this was in the depth regime at which the decision to dive or not dive would be made.

Based on the calculated propagation loss and the known transmission level and beam pattern, the RL_rms_ at every range hydrophone was calculated for every ping transmission (1 second duration). These data were divided into 30 minute segments, this being the approximate amount of time over which a group of beaked whales produces clicks during a deep foraging dive [18]. The maximum modeled RL_rms_ for every range hydrophone in every 30 minute segment before and during MFA operations were determined. GVP start times and their associated center hydrophones were also recorded for each segment. These data were correlated with the times of MFA use to provide a record of the maximum sonar RL_rms_ and the presence or absence of a GVP start for every half hour time segment on every range hydrophone. Both the calculated RL_rms_ along with the dive start data for each 30 minute segment are hosted at the Naval Undersea Warfare Center in Newport Rhode Island and have been cleared for release upon request.

### Analysis

A Generalized Additive Model (GAM, [29]) was used to model the presence or absence of GVP starts centered on each hydrophone and for each 30 minute segment, as a smooth function of the maximum RL_rms_, using a binomial distribution with a logit link function. Analyses were performed using the mgcv library (version 1.7-22) within the software R (version 2.15.2; [30]). The smooth function was specified using a thin plate regression spline, the default in the mgcv library; results were not sensitive to choice of smoother. The model assumes that each GVP start is independent (given the RL_rms_); therefore residuals were checked and no temporal or spatial autocorrelation was evident.

The fitted GAM was used to predict the probability of a GVP start at a range of RLs. To translate this relationship into a risk function, the estimated baseline probability (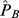) of a GVP start when no sonars were transmitting was calculated using the data from the 39 half-hour segments in the 19 hour period before the start of the exercise, as follows: 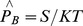
(1)


Where *S* is given by
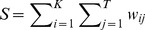
(2)


and w_ij_ is an indicator variable which takes the value 1 if at least a dive occurred at hydrophone *i* (*i* = 1,2,...,82) during the half hour *j* (*j* = 1,2,...,39), and 0 else otherwise. This corresponds to the empirical estimator of the probability of a dive occurring during the control period, i.e. the observed frequency of periods with dives in the control period, and max(*S*)  =  *KT*, hence this is well defined as a probability.

The probability of disturbance, 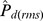, i.e., the change relative to the baseline GVP start rate, at a particular RL_rms_, was then estimated as
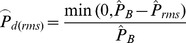
(3)


where 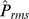 is the GAM-based estimated probability of a GVP start at a given RL_rms_. The min operator is used to ensure that the equation corresponds to a probability (i.e. 0 ≤ 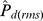
≤ 1). Provided a long enough time period is used and sonar has a negative or no effect on diving behavior, 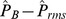 will be equal or greater than 0.

The resulting estimated risk function (i.e., relationship between received level and disturbance) does not have a parametric form, because it is based on the output from a GAM, which is by its nature semi-parametric. To facilitate ready dissemination of the results, an approximating parametric function was derived. The estimated probability of disturbance was modeled as a function of received level using a Generalized Linear Model (GLM) assuming a Gaussian response distribution and a probit link function.

Uncertainty in the estimated probability of disturbance was quantified using a bootstrap procedure. For 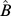, a nonparametric bootstrap was used to generate 10,000 random realizations by resampling with replacement from the 39 segments on each hydrophone in the baseline period. For 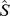, a parametric bootstrap was used, in which 10,000 random realizations were obtained from the fitted GAM using a multivariate normal distribution to generate new parameter estimates for the smooth basis functions, based on the estimated values and variance-covariance matrix [29]. These values were then combined to yield 10,000 bootstrap resampled estimates of 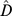. Confidence intervals on 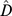 were then computed by taking the 2.5^th^ and 97.5^th^ percentiles of the resulting distribution.

## Results

A total of 106 GVP starts were identified in data collected from 91 hydrophones in the segments before the multi-ship exercise began. These were used to provide an estimate of baseline probability of a GVP start, B, of 0.02893 (95% CI;.02890–0.02896).

**Figure 1:**
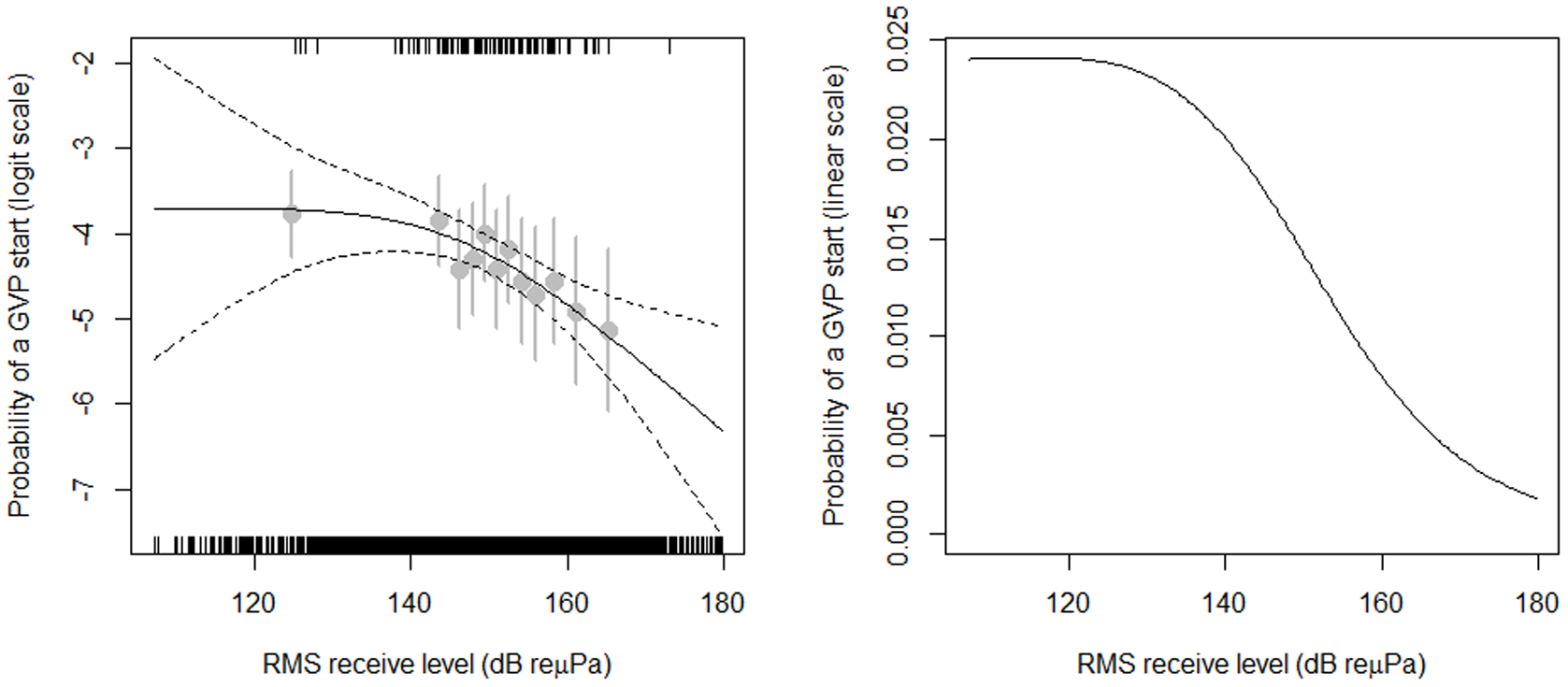
Estimated probability of a GVP start as a function of maximum RL_rms_ in a 30 minute segment on a given hydrophone on the logit (left plot) and linear (right plot) scale. Dashed lines indicate pointwise 95% confidence limits on the fitted relationship. Short vertical lines at the top and bottom of the plots show the data used in the model: those at the top indicate the RL_rms_ where GVP starts were observed, while those at the bottom of the plots indicate RL_rms_ where GVP starts were not observed. The grey dots provide a summary of these data, and can be used to assess the goodness-of-fit of the fitted relationship – they are the proportion of the data where a GVP start was observed, each calculated using approximately 1/12^th^ of the data going from lowest to highest RL. Grey vertical lines indicate 95% binomial confidence intervals on these proportions.

During the six sonar scenarios, 105 dive starts were identified. The GAM fit estimated the probability of a dive start, *S_rms_*, for a given received level declined from less than.0238 at a received level of 110 dB (the lowest received level during an operation) to ∼.0019 at a received level of 180 dB (the maximum received level). The fit of the model to data was excellent (Figure 1).

**Figure 2:**
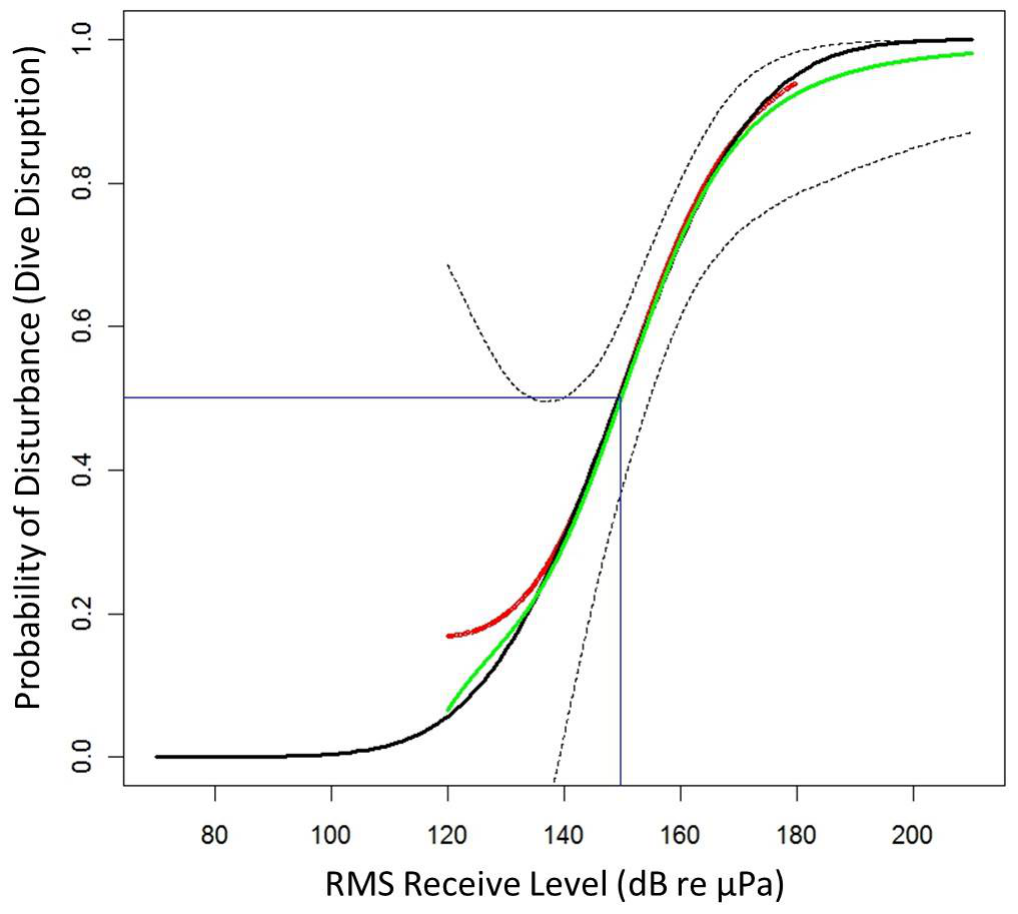
The probability of disturbance (*D_rms_*) as a function of sonar RL_rms_. The GAM fit to the recorded data is shown in red with the bootstrap mean shown by the green with the point-wise 95% confidence limits indicated by dotted lines from the bootstrap. The parametric GLM approximation is shown in black. There is a.5 probability of disturbance at a RL_rms_ of 149.8 dB; this is indicated in blue.

These results were combined using Equation 3 to calculate a series of estimates of disturbance, 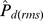, at each received level as given in Figure 2 (red line). The resulting curve show a ∼.95 probability of disturbance at an RL_rms_ of 180 dB and a ∼.2 probability of disturbance at 130 dB, keeping in mind the wide confidence intervals at low received levels. These wide intervals resulted from the high source levels during operations that in turn resulted in few exposures at these lower levels within the field of hydrophones.

The GLM fit was an excellent approximation to the GAM (Figure 2), and has the advantage of being easy to represent in parametric form: 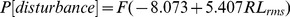
(4)


where *RL_rms_* is the receive level and *F(z)* is the cumulative normal distribution function [31].
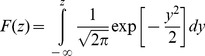
(5)


## Discussion

**Figure 3:**
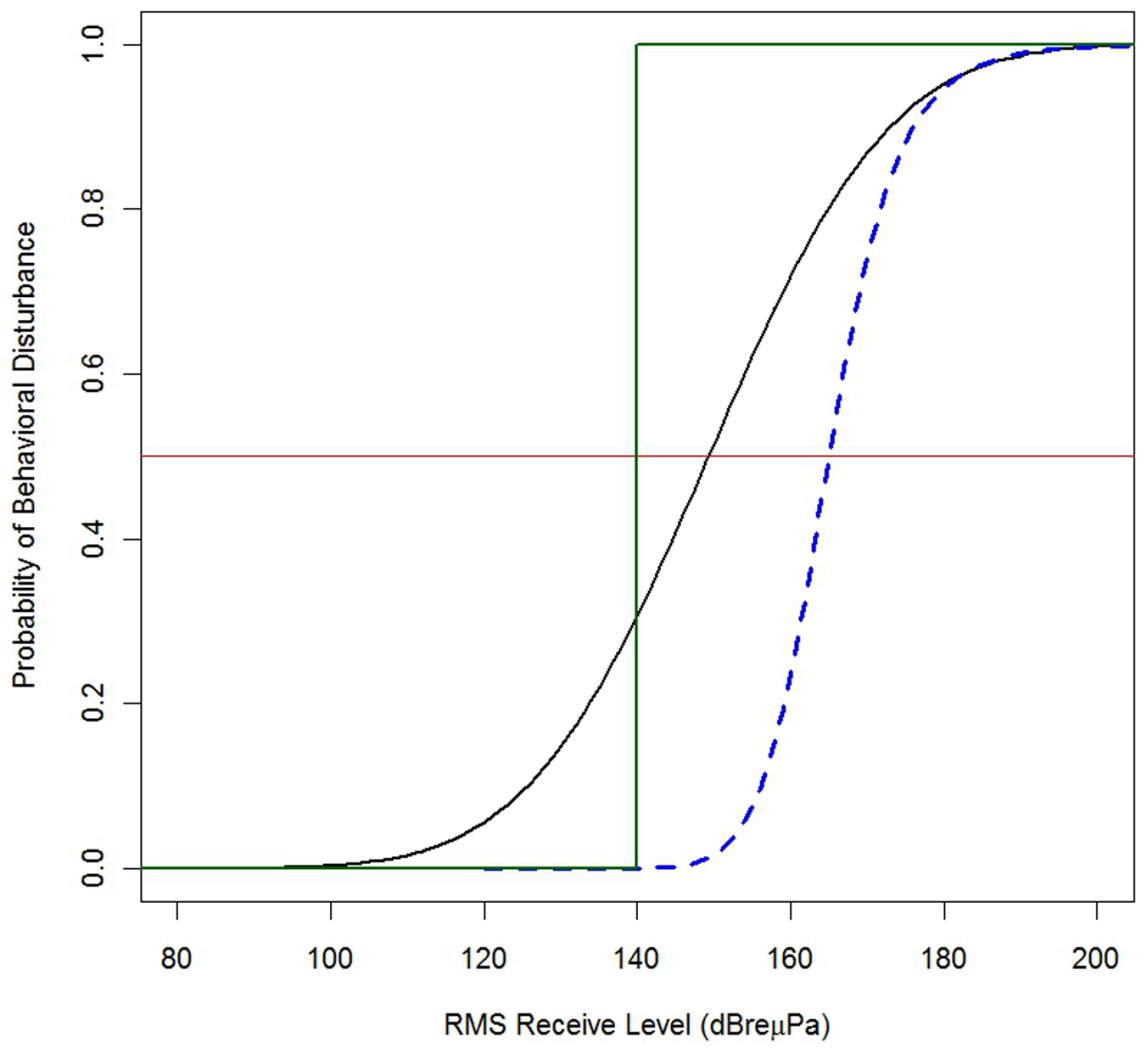
A comparison of risk functions relating the probability of disturbance to received level for beaked whales exposed to sonar signals. The current step function used by the U.S. Navy is shown by a green line and the historical function by a blue-dashed line. The empirical function developed in this paper is shown by a solid black line. A solid red line marks the.5 probability of disturbance.

We have derived an empirical risk function for beaked whales that relates the probability of behavioral change to the RL_rms_ from MFA sonar. This is compared to risk functions that have been used previously in Figure 3.

The empirical risk function predicts that there is a 0.5 probability of disturbance at a received level of 150 dB (CI: 144–155) whereas the historical function predicts this will occur at a received level of 165 dB and the step function currently used by the U.S. Navy assumes that a response is certain at any received level above 140 dB. This suggests that use of the historical function would lead to an under-estimate of the effects of an operation using sonar on beaked whales, whereas the current step function would over-estimate the effects.

The derivation of the risk function was limited to data on RLs above 125 dB because of the limited extent of the hydrophone field and the high source levels of the sonars deployed during the operation. Therefore, the confidence intervals for received levels below approximately 135 dB are very wide. As data for lower level sources, such as dipping helicopter sonar, become available, it may be possible to reduce the uncertainty associated with the probability of a behavioral response at lower received levels.

During these military operations, multiple sound sources and source types were in use. Often, these transmissions were coincident. In this paper we considered only the maximum RL_rms_ recorded from any source within each 30 minute segment. Consequently, the loudest sonars, in the 3–4.5 kHz range dominated the levels recorded. We did not address the potential for cumulative effects from multiple sources operating simultaneously or close together in time. These additional sources may have exacerbated the animals’ reactions and caused them to alter their behavior at a lower received level as compared to their reaction to a single source.

To date, experiments involving the playback of sonar-like sounds to beaked whales have used a portable sound generator with a source level significantly less than typical U.S. Navy sonar [6]. To achieve the desired received level, the source was positioned within 3 km of the experimental animals. As a result, these playback experiments were not able to account for the effect of distance from the source on response. By contrast, we used RL_rms_ levels derived from actual navy operations so that the RLs were directly related to the beaked whale’s distance from the MFA sonar.

The risk function was derived by isolating groups of foraging *Md* using passive acoustics. We are not able to ascertain group composition based on these data so there is no way to determine if the results are a function of such factors as animal age or sex, or group composition or size.

The risk function we have derived does not address the issue of how behavioral disruption may affect the overall health of a beaked whale population. Previous studies strongly suggest animals move off range in reaction to sonar and return after the cessation of operations [18], [16], [17], but the total time over which foraging is disrupted is unknown. If the animals move off the AUTEC range and resume foraging soon after, such behavioral changes may have little effect. However, if prey availability off-range is poor and the duration of displacement is long, net energy intake may be diminished, even if the animals continue to forage. Thus, the cumulative effect of extended disturbance on total energy balance could result in diminished body condition of some mature females, which could have consequences for their reproductive success through multiple developmental stages from initial pregnancy, to lactation, and up to the time of calf weaning. Such negative effects in turn could result in reduced calf survival and longer inter-calf intervals, ultimately resulting in lower reproductive rates.

Ongoing research has provided estimates of *Md* density in the TOTO [32], [18]. The risk function provides a means of predicting the probability of disruption on an exercise-by-exercise basis. AUTEC data provide a record of MFA active operations throughout the course of a year. In addition, data from satellite tagged *Md* [17] are providing insight into the effect of sonar disruption on foraging behavior over longer time scales. By combining these data sets, the cumulative effect of repeated sonar exposure can be estimated in terms of the total number of foraging dives lost. For *Md*, total caloric intake is directly related to the number of foraging dives they make, and these occur at a known rate. A simple energetics model could therefore be used to translate lost dives into an estimate of total energy loss. This loss could then be used to predict changes in maternal fitness, thus providing insights into the consequences of behavioral change for long-term population health.
