# Correction: A Risk Function for Behavioral Disruption of Blainville’s Beaked Whales (*Mesoplodon densirostris*) from Mid-Frequency Active Sonar

**DOI:** 10.1371/journal.pone.0116555

**Published:** 2014-12-22

**Authors:** The PLOS ONE Staff

There is an error in Equation 4. Please see the corrected Equation 4 here.
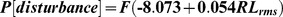
(4)


There is an error in the legend for Figure 2. Please see the complete, corrected Figure 2 here.

**Figure 2:**
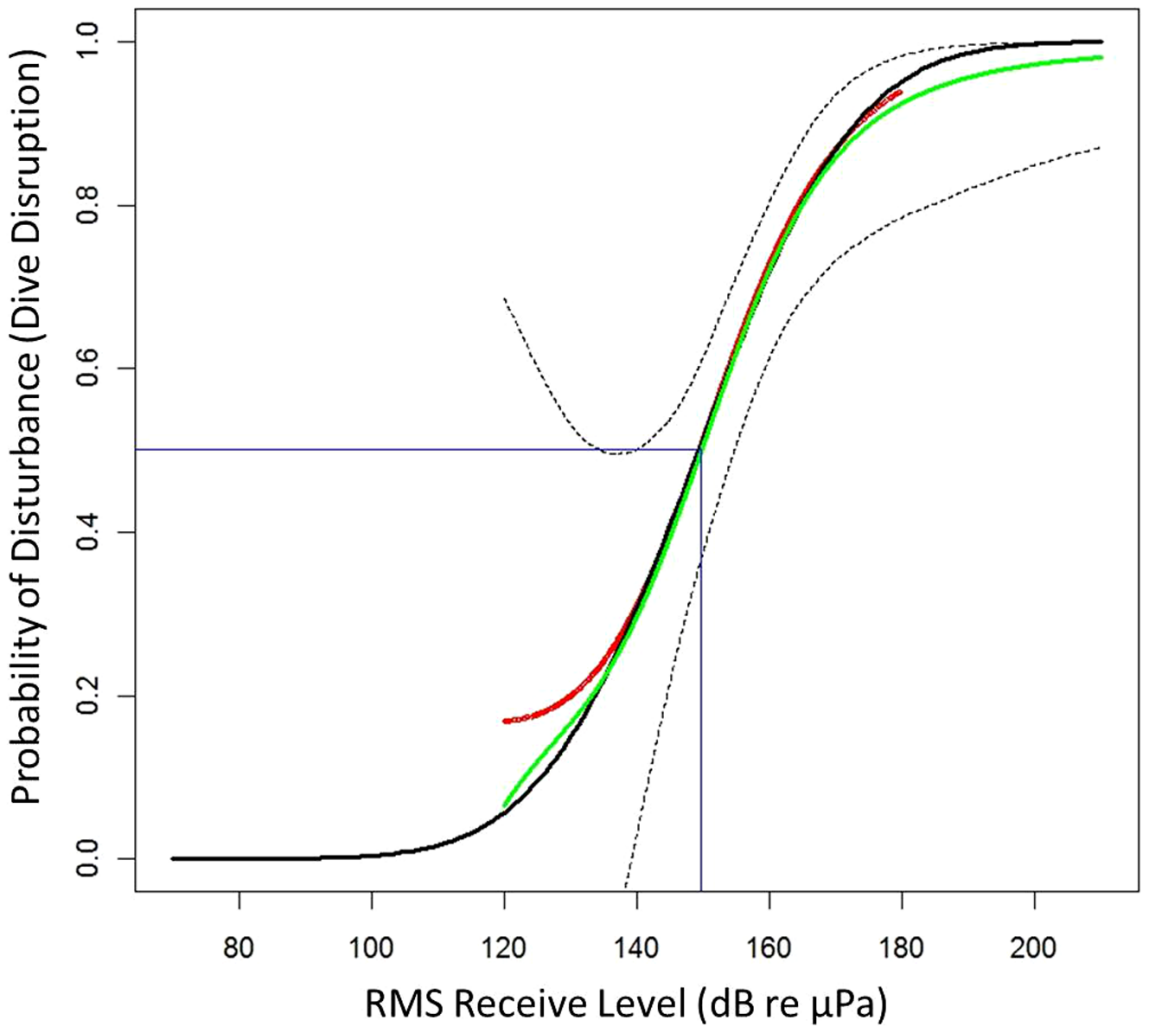
The probability of disturbance (D_rms_) as a function of sonar RL_rms_ . The GAM fit to the recorded data is shown in red with the bootstrap mean shown by the green with the point-wise 95% confidence limits indicated by dotted lines from the bootstrap. The parametric GLM approximation is shown in black. There is a.5 probability of disturbance at a RL_rms_ of 149.5 dB; this is indicated in blue.
